# Parabens and estrogen-mediated cancers: unraveling coincidence, co-occurrence, and causality

**DOI:** 10.1038/s44294-026-00162-7

**Published:** 2026-08-28

**Authors:** Zehra Esra Ilhan, Andrea Marchetto, Georgia Lorentzen, Paweł Łaniewski, Leslie V. Farland, Melissa M. Herbst-Kralovetz

**Affiliations:** 1 Université Paris-Saclay, INRAE, AgroParisTech, Micalis Institute, Jouy-en-Josas, France; 2 Department of Obstetrics and Gynecology, College of Medicine-Phoenix, University of Arizona, Phoenix, AZ USA; 3 Department of Metabolism, Digestion and Reproduction, Division of Systems Medicine, Imperial College London, London, UK; 4 Department of Basic Medical Sciences, College of Medicine-Phoenix, University of Arizona, Phoenix, AZ USA; 5 Department of Epidemiology and Biostatistics, Mel and Enid Zuckerman College of Public Health, Department of Obstetrics and Gynecology, College of Medicine -Tucson, University of Arizona, Tucson, AZ USA

**Keywords:** Cancer, Oncology

## Abstract

Paraben esters are endocrine disruptors linked to breast cancer, yet their role in other estrogen-mediated cancers remains limited. Preclinical evidence suggests that parabens accumulate in tissues and may promote carcinogenesis, while clinical data indicate widespread exposure. Our re-analysis of cervicovaginal metabolomes revealed elevated paraben metabolites in endometrial and cervical cancer patients compared with controls. Overall, longitudinal studies considering intrinsic and environmental factors are needed in assessing paraben-related cancer risk.

## Introduction

Hormone-dependent cancers are increasingly becoming more prevalent worldwide, with higher diagnosis rates in high-income countries and higher mortality in low- to middle-income countries^1^. Estrogens are particularly involved in the development of endometrial, ovarian, and breast cancers^2^, and are implicated in the progression of cervical cancer in women^3^ and prostate cancer in men^4^. The surge in the incidence of estrogen-mediated cancers parallels increased exposure to synthetic or natural compounds that mimic the activity of estrogens, known as endocrine disrupting compounds (EDCs). EDCs have been associated with high body mass index^5^, which has also been associated with increased risk of endometrial^6^ and breast^7^ cancers. Among the EDCs, paraben esters constitute a large group of chemicals commonly found in food, personal care and hygiene products, and other everyday items.

Parabens have been associated with several cancers, more specifically with an increased risk of breast cancer^8,9^, and have been observed to accumulate at higher levels in cancer tissue compared to healthy tissue^8^. Moreover, in vitro and ex vivo models have provided mechanistic insights into cancer-associated adverse outcomes of parabens^10,11^. These compounds exert estrogenic effects by activating estrogen receptors, enhancing the production or actions of natural estrogens, and increasing circulating estrogen levels by inhibiting sulfotransferase enzymes that are responsible for the excretion of estrogens from the body^12^. Despite the evidence of estrogenic activity and detection of parabens in certain cancer tissues, the relationship between parabens and estrogen-mediated cancers remains poorly understood due to the complex contribution of various intrinsic and environmental cofactors, inter-individual differences, and the rather chronic nature of health effects of these molecules. It is unclear whether these associations are merely coincidental, indicative of a causative role, and/or whether the cancer is a consequence of paraben exposure. Moreover, health disparities in exposure patterns and mechanistic links are not well understood.

In this review, we provide our insights on parabens and estrogen-mediated cancers by presenting an overview of clinical studies concerning cancer patients and individuals with non-cancerous conditions (i.e., infertility, endometriosis, etc.), utilizing targeted or untargeted metabolomics approaches. Additionally, we present novel data on parabens detected in the local cervicovaginal microenvironment of patients newly diagnosed with cervical or endometrial cancer. We discuss the factors impacting the exposure patterns and summarize findings from cell/tissue culture and animal models to highlight potential mechanisms of action. Our focus within the scope of this review is primarily on cancers affecting women’s health.

## Results

### Circumstances leading to increased exposure to parabens in the context of estrogen-mediated cancers

Since their introduction in the 1950s as preservative agents, paraben molecules have been increasingly incorporated in the formulation of everyday products to extend the shelf life of cosmetics, pharmaceuticals, food and drinks, and various personal care products (PCP) including sunscreen, makeup, toothpaste, shampoo, and feminine hygiene products^13^. Epidemiological studies investigating the association between PCP use and cancer risk have yielded inconsistent results. For example, PCP use during puberty (*n* = 45,465) has been associated with an increased risk of breast cancer^14^, whereas a prospective cohort study (*n* = 106,978) did not find any association between PCP and breast or endometrial cancer risk^15^. However, these studies largely rely on self-reported product use and do not account for the specific chemical constituents of PCPs, limiting their ability to identify associations with individual compounds. Regardless, the widespread use of these products combined with the physicochemical characteristics of the parabens has led to their accumulation in soil, water, and air^16^, consequently contaminating food and drinks and potentially causing indirect exposure. Collectively, this may exacerbate the health threat and require “One Health” approaches that would allow more comprehensive characterization of potential risks and exposure routes.

**Fig. 1:**
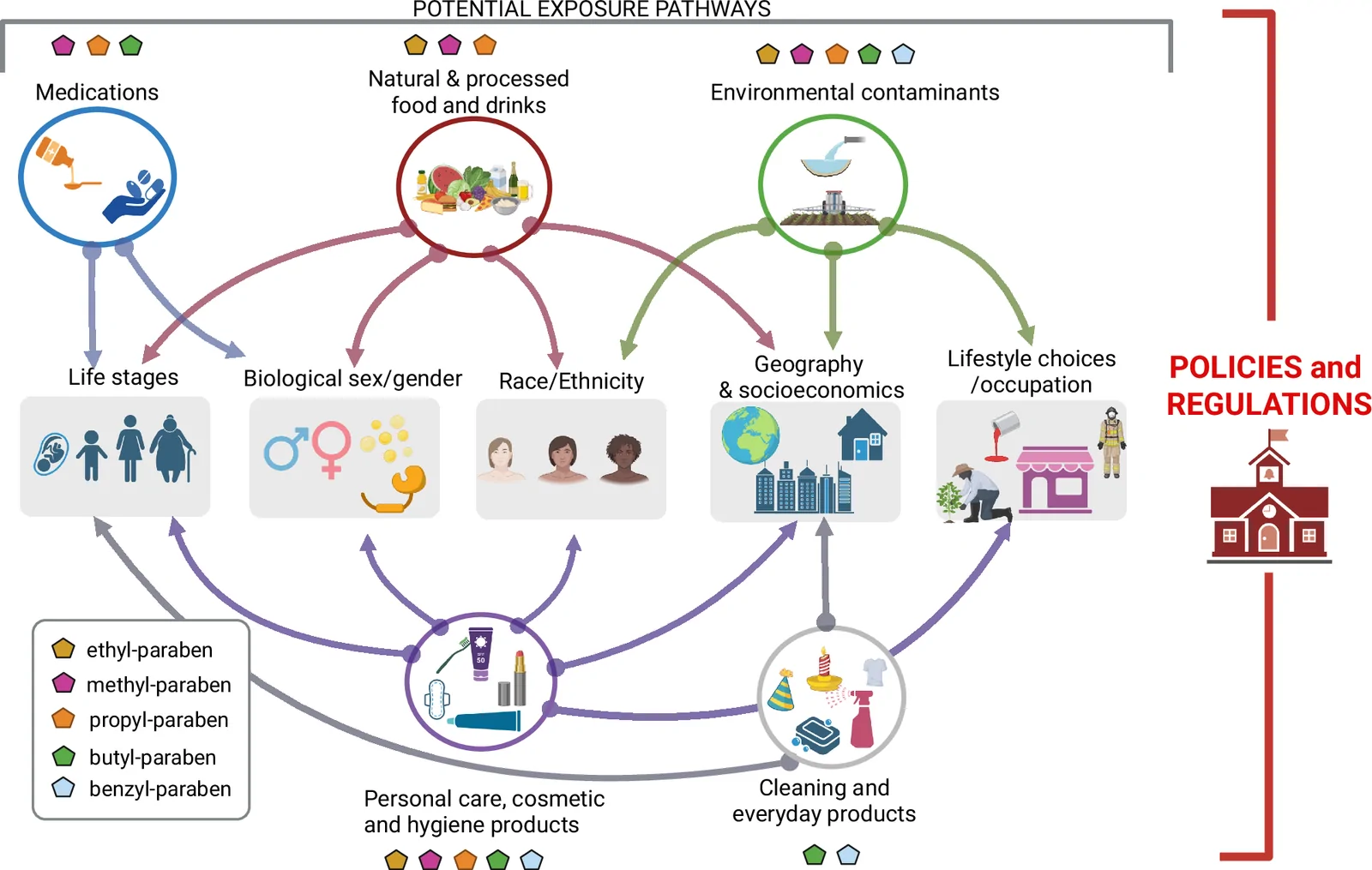
Factors influencing exposure to parabens. Exposure patterns to paraben esters are impacted by several factors including the product availability, personal choice and indirect exposure via contaminated food, water, and environment. Policies, regulations, and demographics may mediate the exposure patterns and levels. Created in BioRender. Herbst-Kralovetz, M. (2025) https://BioRender.com/izjjp8z.

We highlight several factors and pathways that need to be considered when evaluating paraben exposure and the increased risk of developing estrogen-mediated cancers (Fig. 1). We identified life stages, biological sex/gender, ethnicity/race, geography and socioeconomics, lifestyle and dietary choices and occupation, all of which influence direct exposure (via product availability, consumer preferences) or indirect exposure (via contaminated environment, food, and water). For example, geography and socioeconomics strongly shape environmental exposure, product availability, consumption patterns, and advertisements. Geography also influences policies and regulations, which are country- or region-specific. These regulations determine daily intake limits, maximum allowed concentrations, and the type of paraben molecules in various products. For example, in the European Union (EU), several types of parabens are banned (i.e., phenyl, benzyl), and others (i.e., methyl-paraben) are only allowed in cosmetic products up to 0.4% individually or 0.8% as total parabens^13^. Conversely, in the USA, there has not been a ban on parabens in cosmetics yet based on Food and Drug Administration (FDA) regulations; however, there are limits for food items, with the maximum allowed concentration capped at 0.1%^16^. The regulations for cosmetic ingredients that contain parabens vary by country as well. For example, South Africa, which has the largest cosmetics market in Africa, aligns with the EU for the cosmetics regulation frameworks, whereas India has adopted a framework similar to the USA, including some features of the EU^17^.

Prior research assessing paraben exposure based on cross-country analysis, through urinary measurements, reported that exposure was much greater in China and Korea than in Europe and United States^18^, indicating widely varying product use, regulations, and ultimately health effects in different geographical regions. Driven by regulatory efforts, consumer advocacy, education, research, and the influence of social media, there has been a rise in the “clean” product movement - particularly in the development of paraben-free formulations. However, replacement or substitute compounds may also possess unintended toxicological or sensitization-related effects. Therefore, the safety of alternative compounds also should be comprehensively evaluated to avoid “regrettable substitution”, where one potentially harmful compound is replaced with another insufficiently characterized alternative, as previously comprehensively reviewed^19^.

Besides geography and socioeconomics, interpersonal differences, biological (genetics, sex), social (race/ethnicity) or behavioral (life choices) factors play a key role in determining the exposure levels, frequency, and the specific subtypes of parabens an individual is exposed to. Lifelong exposure begins during the prenatal period, and highest exposure has been predicted to occur during the reproductive years and pregnancy in women^20^. Typically, women use more PCPs, cosmetics, and hygiene products than men^21^. In line with this, product use is also associated with race/ethnicity, as non-White women might be more exposed to parabens than White women^20^, potentially due to use of hair and skincare products specific for Black and Hispanic/Latina women^22^. A recent study highlighted higher blood concentrations of parabens in women than men across various occupational categories^23^. Occupations can alter paraben exposure, with women working in the beauty industry having greater urinary concentrations of paraben esters^24^ than those employed in other fields.

Given that inter-individual variations that are shaped by biological, social, behavioral and environmental factors greatly influence levels, types and frequency of paraben exposure, they need to be taken into account when assessing the potential link between parabens and the development of estrogen-mediated cancers. Additionally, the relative impact of direct versus indirect exposure as well as acute versus chronic exposure on cancer risk remains uncertain. Understanding these exposure patterns is critical for developing effective cancer risk-reduction strategies and assessing public health risks, as these pathways may reflect the rising trends in estrogen-mediated cancers.

### Co-occurrence of parabens and estrogen-mediated cancers

With the advancements in the field of metabolomics, parabens have been increasingly detected in various biological fluids and tissues using both targeted and non-targeted approaches in healthy individuals^9^ and cancer patients^8,9,25,26^. The majority of cancer studies have focused on breast cancer, highlighting a lack of data on parabens and other estrogen-mediated cancers such as ovarian and endometrial cancer.

The associations between cancer risk and parabens have often been established based on concentrations of paraben esters in specified biospecimens (summarized in Table 1), including studies reporting greater abundance or detection of parabens in cancer tissues or other samples from patients with cancer compared to controls, as well as epidemiological analyses calculating odds ratios from biospecimen measurements. These studies were mainly conducted in the USA; however, there were a few studies from Türkiye^25^ or Iran^8^. In breast cancer patients, odds ratios were statistically significantly higher for urinary methyl- and butyl-paraben esters and concentrations of paraben esters were significantly greater in the malignant breast tissue versus benign breast tissue^8^. Similarly, parabens were detected in higher amounts in endometrial cancer tissue in comparison to premalignant endometrial intraepithelial neoplasia^25^. Interestingly, history of self-reported uterine cancer was inversely associated with ethyl-paraben^27^.

**Table 1:** Detection of paraben in serum, urine, and tissue of cancer patients

Cancer type	Time of sampling	Population size (*n*)	Age [range, (average or median)]	Location, race/Ethnicity	Sample type	Analysis method	Type of paraben and concentration range	The association between parabens and cancer
Breast								
Breast^26^	∼3.3 years before diagnosis	1032 cancer cases & 1030 age-matched controls	45–75 (post-menopausal)	USA/ African American Latino Native Hawaiian Japanese American White	Urine	LC-HRAM-MS	Average (μg/g creatinine) MeP 49.4 PrP: 18.5 EtP: 1.15 BP: 0.15 BeP: 0.004	Inverse association with total parabens: OR_T3_ vs _T1_ = 0.77 (95%CI: 0.62–0.97), *P*_trend_ = 0.03
Breast^9^	Within 3 months of diagnosis	708 cancer cases & 598 controls	NA - 65 (34.2)	USA/ not specified	Urine	HPLC-MS	Total range (μg/g creatinine) MeP: (1-3174) PrP: (DL < to 3116) BP: (DL< to 173)	MeP and BP associated with increased odds of breast cancer. Increased risk in hypomethylated global DNAm group (OR:1.46)
Breast^8^	Newly-diagnosed	101 cancer cases & 47 benign controls	13–76 (41.5)	Iran	Tissue (breast)	GC-MS	(metastatic-benign, ng/g) MeP: (173–149) EtP: (122–102) PrP: (93–59) BP: (82–45)	Significantly higher butyl and propyl (*p* = 0.001) in metastatic (cancer) vs benign (fibroadenoma) tumors
Breast^43^	Diagnosed	53 cancer cases	36 non-menopausal 16 menopausal	Iran	Tissue (breast)	Targeted GC	(benign -malignant, ng/g) MeP: (164–178) EtP: (144–136) PrP: (97–99) BuP: (107–87) Total: (507–497)	Positive dose response pattern for MePB (*p* < 0.001)
Breast^80^	History of cancer (timing not available)	134 cancer & 4321 cases	47.52 (16.98) for healthy and 65.29 (12.84) for cancer patients	USA/Mexican American, Hispanic African American	Urine	HPLC-MS	Median (IQR, Range ng/mL) MeP: 4.86 (2.29, 3.62–5.91) EtP: 0.64 (2.94, -0.35–2.6) PrP: 3.19 (2.89, 1.55–4.44) BP: -1.61 (2.65, -1.96–0.69)	No statistically significant association
Endometrial / Uterine cancer								
Endometrial^41^	+10 years before diagnosis	139 pre-cancer cases and 139 cancer-free controls	59-69 (62) – at the time of urine collection	USA/ White, African American, Native Hawaiian, Japanese American, Latina	Urine	HPLC tandem mass spectrometry	Median creatinine normalized (control-cases) MeP: (98.73–78.64) EtP: (3.23–1.47) PrP: (20.16–11.30) BP: (0.36–0.15) Total: (137.13–11.55)	No statistically significant association
Endometrial^25^	Concurrent with diagnosis	33 cancer cases & 6 controls (intraepithelial neoplasia)	49–57	Türkiye	Tissue (endometrial, myometrial)	UHPLC-MS/MS	(ng/g) MeP: < DL EtP: < DL PrP: 6.3–60.5 BeP: 9.1–22.4 IsoBP: 5.4–118.2	Higher in endometrial carcinoma tissue compared to normal endometrium (*p* = 0.02)
Uterine^27^	History of cancer (timing not available)	37 cancer survivors & 3432 control	>20 (48)	White (non-hispanic) Black (non-hispanic) Mexican American Other Hispanic Other	Urine	HPLC-Tandem MS	NA	Higher EtP inversely associated with previous history of cancer OR:0.31, 95% CI: 0.12, 0.85

Parabens have also been investigated in the context of non-estrogen-mediated cancers. For example, greater concentrations of methyl, ethyl, propyl, and butyl parabens were detected in the urine of lung cancer patients than in non-cancer controls^28^. Notably, risk for lung cancer is 2.7 times higher in women than men^29^. Parabens in urine have been associated with self-reported prostate cancer^30^, thyroid cancer^31^, and previous melanoma diagnosis^27^. However, these cancers are also strongly influenced by multiple intrinsic and extrinsic risk factors, including smoking habits, genetic susceptibility, hormonal status, medication use, and other environmental exposures. Hence, parabens should be considered as one potential component within a complex network of interacting factors rather than as isolated drivers of cancer development. Even though these cancers are not widely recognized as being directly driven by estrogen signaling, parabens may also induce carcinogenic pathways via non-estrogen-dependent mechanisms. Additionally, parabens have also been detected through untargeted metabolomics in bladder and colon cancer studies^32,33^; however, they have not been prominently analyzed within the context of cancer risk, as most studies focused on intrinsic metabolites. Besides cancer, parabens have been frequently associated with the pathophysiology of other gynecological and reproductive disorders (Table 2). For example, early menopause^34^, female infertility^35^, endometriosis^36^, fibroids^37^ and ovarian aging^38^ have been positively associated with at least one type of parabens. Many of these reproductive conditions, including ovarian aging and menopause, are also risk factors for estrogen-mediated cancers^39^.

**Table 2:** Detection of paraben esters (MeP, methyl-paraben; PrP, propyl-paraben; EtP, ethyl-paraben; BP, butyl-paraben; HeP, heptyl-paraben) in urine samples of obstetric/gynecological conditions other than cancer

Condition	Time of sampling	Population size (*n*)	Age [range, (average or median)]	Location, race/Ethnicity	Sample type	Analysis method	Type of paraben and concentration range	The association between parabens and the condition
Ovarian aging/fertility^38^	Before fertility treatment	192	21–46.7 (36.1)	USA/Caucasian, African American, Asian	Urine	HPLC-MS	(μg/L) MeP: 3-4400 PrP: <DL – 1430 BP: <DL - 142	Positive correlation between PrP and diminishing ovarian reserve (*p* = 0.007)
Midlife-reproductive health outcomes^34^	Not available (Premenopausal and perimenopausal women)	101	45–54 (47)	USA/non-Hispanic White	Urine	HPLC-MS	BP < EP <PrP < MP	Positive association between total parabens and hot flashes, FSH, reduced ovarian volume
Endometriosis^36^		33	20–54 (38)	Spain	Urine	UHPLC-MS/MS	Mean (ng/mL) MeP: 236.56 EtP: 44.20 PrP: 16.43 BP: 0.81	BP and PrP positively associated with the key genes for the development and proliferation of endometriosis.
Fertility^81^	During the enrollment before conception	525 couples	29.2 + 3.2	China	Urine	Targeted HPLC-MS/MS	MeP: (95.7–79.9) EtP: (1.78– 1.11) PrP: (2.6– 3.27) BP: (0.02– 0.11) HeP: LOD	PrP, BP, and HeP associated with reduced fecundability and increased risk of infertility

Our review demonstrates that our understanding of parabens in cancers, specifically estrogen-mediated cancers, is still largely limited. Therefore, there is an urgent need to characterize parabens in various biological matrices, including those reflecting localized exposure (e.g., tissues and lavages) and systemic circulation (e.g., blood, urine, and feces), across different types and stages of estrogen-mediated cancers, particularly in understudied reproductive tract organs. Additionally, banked biological samples can be evaluated to better understand exposure pathways and their relationship to cancer progression.

### Complexities of studying paraben exposure and cancer in cohorts

Associating parabens with cancer risk poses several challenges, including sampling time, type of sample, and heterogeneities in the populations due to the presence of several cofactors. The majority of prior research on cancer (summarized in Table 1) relied on the targeted detection of parabens mostly in urine or tissue either before, during, or after diagnosis, based on a measurement at a single timepoint. Time of sampling is critical for several reasons. Studies utilizing pre-diagnostic sampling primarily assess whether exposure precedes disease development, whereas studies performed at the time of diagnosis or in tumor-associated tissues mainly reflect co-occurrence within the local environment. In contrast, retrospective survivor studies may additionally reflect disease-associated behavioral, metabolic, or pharmaceutical changes following diagnosis. For example, sampling of current cancer patients suggests possible heavy medication use by the patients due to therapies which might exacerbate exposure levels, as parabens are common drug excipients. Sampling too far in advance or after diagnosis in cancer-free patients may complicate the interpretation of the relationship between exposure and cancer risk, as parabens are rapidly metabolized (<24 h) and excreted in urine^40^. To establish a more accurate association, longitudinal sampling reflecting chronic exposure history is necessary. For example, surprisingly, an inverse association between paraben exposure and breast or endometrial cancer was observed when sampling was performed in individuals before cancer development^26,41^. In these studies, the cancer diagnosis occurred on average 6.6 years after urine sampling in the endometrial cohort^41^ and 5.5 years after in the breast cancer cohort (sampling 2001–2006 and diagnosis 2001–2014)^26^. Whereas a positive association between parabens and breast cancer, with a higher odds ratio, was observed when sampling was conducted either at the time of the diagnosis or 3 months after the diagnosis^8,9^.

Biospecimen type is also crucial for studying these associations. As parabens are excreted in urine, these samples indicate daily or relatively recent exposure due to the rapid metabolism and excretion of parabens, whereas tissue-based measurements may provide insight into local accumulation or tissue-specific exposure patterns over longer durations of time. Measurement of paraben esters in the local breast cancer microenvironment revealed an interesting phenomenon: the concentration of paraben esters was statistically significantly higher in malignant tissue compared to adjacent healthy tissue^8^. This co-occurrence may reflect altered tissue metabolism, differential accumulation of paraben molecules, disease-associated changes within the tumor microenvironment, or a potential role of parabens in tumorigenesis, the mechanisms of which need to be elucidated. A pharmacokinetics study in rats revealed that oral or topical exposure to parabens leads to mainly excretion via urine (70%) or feces (4%) but also accumulation in tissues (2%)^42^.

The heterogeneities in exposure pathways and inter-individual variations present additional challenges in associating parabens with an increased cancer risk. For example, women with global DNA hypomethylation may have a greater risk of breast cancer when exposed to parabens. A dose-response relationship with breast cancer risk factors (e.g., ER+ and PR+) was also reported; methyl-paraben and total paraben concentrations were positively associated with these risk factors^43^. Targeted metabolomics studies that focus exclusively on parabens often overlook the influence of other potential EDCs or intrinsic factors like estrogens and the gut microbiome^44^. These factors are closely linked to activation of the nuclear receptors that parabens also target^45^. Without comprehensive metadata accounting for other confounding factors (i.e., exposure to carcinogens, estrogenic compounds, and other xenobiotics), these associations remain difficult to interpret. On the other hand, detection of parabens may potentially reflect broader environmental or lifestyle-associated exposure patterns to EDCs, although validation studies are needed. Co-exposure to other environmental and intrinsic factors should also be considered in terms of cancer risk, as they may all individually or in combination modulate carcinogenesis at multiple stages including initiation, promotion, progression, and invasion.

### Evidence of paraben metabolites in cervicovaginal microenvironment at the onset of endometrial and cervical cancer

**Fig. 2:**
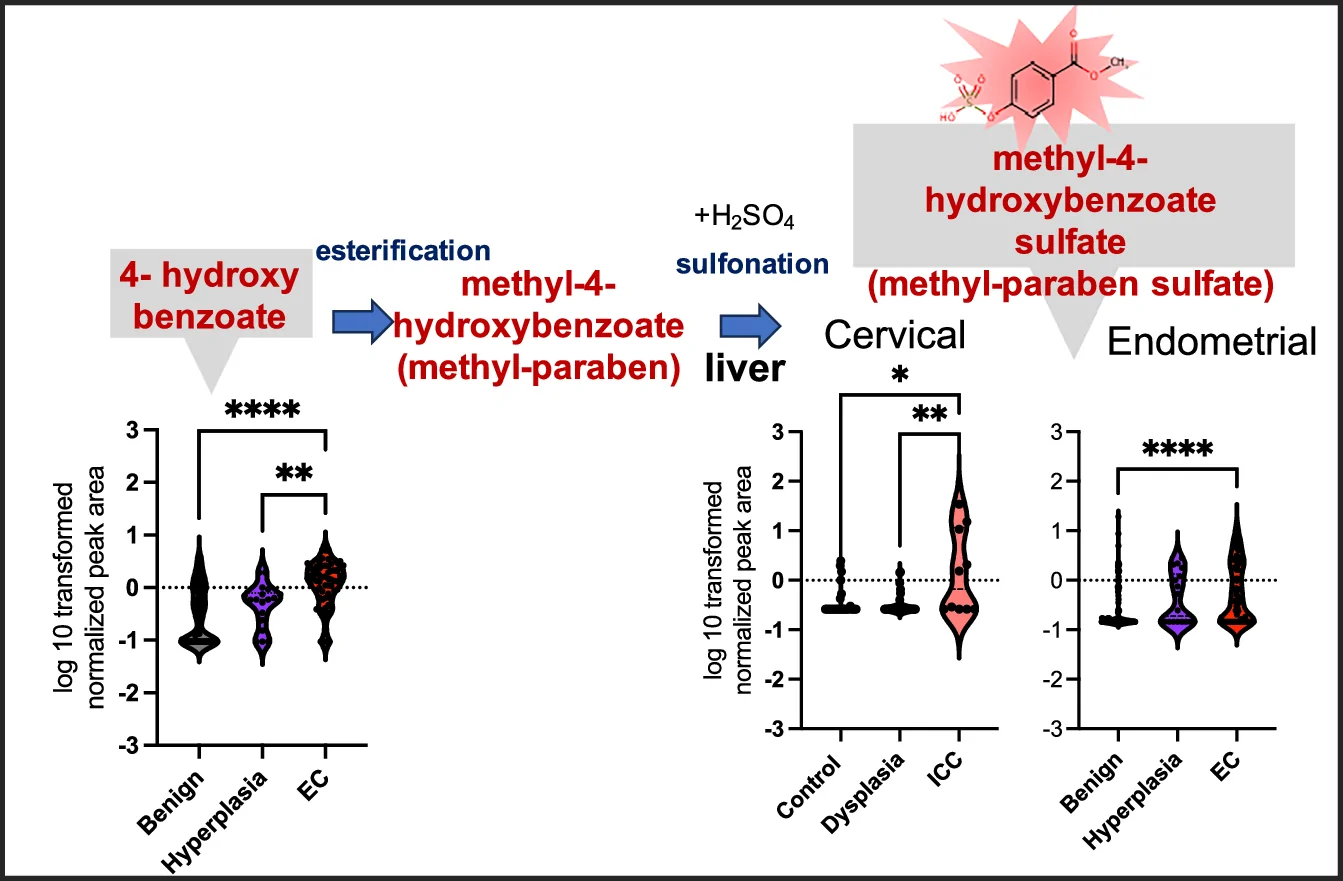
Detection of paraben esters in cervicovaginal lavages in cervical and endometrial cancer cohorts. A liver metabolite of methyl-paraben, methyl-4-hydroxybenzoate sulfate was detected at significantly greater levels in cancer patients [both endometrial (EC) and cervical (ICC)] in comparison to their relative controls. ANOVA, ^*^p < 0.05, ^**^p < 0.01, ^***^p < 0.001, ^****^p < 0.00001.

Parabens have been most commonly analyzed in systemic biospecimens such as plasma and urine, as well as in neoplastic tissues, while their presence within local microenvironments remains less explored. Previously, using global metabolomic approaches, we characterized the metabolome of cervicovaginal lavages from women newly diagnosed with cervical^46^ and endometrial^47^ cancers. This non-invasive sampling approach demonstrated that, with the available library of compounds, 12 benzoate metabolites were detectable in samples from the cervical cancer cohort and 20 benzoate metabolites in samples from the endometrial cancer cohort. The main paraben metabolite, 4-hydroxy-benzoate, was at significantly greater levels in endometrial cancer patients than those with endometrial hyperplasia (precancerous conditions) (*p* < 0.01) or benign controls (*p* < 0.0001). This metabolite was not included/detected in the cervical cancer study. However, it is important to note that 4-hydroxybenzoate can naturally occur in food, particularly berries^48^, and therefore may not exclusively reflect paraben metabolism but also dietary patterns. In both studies, methyl-4-hydroxybenzoate sulfate was detected at significantly higher levels in cancer patients compared to non-cancer controls or dysplasia/hyperplasia patients (Fig. 2, and Supplementary Table 1). Methyl-hydroxybenzoate sulfate is the sulfonated form of methyl-paraben, one of the most used parabens in various products ranging from foods to PCPs. Liver metabolism by sulfatases makes methyl-paraben water soluble by adding a sulfate group, which facilitates its excretion from the body. The greater abundance of methyl-paraben sulfate in cervicovaginal lavages of cancer patients may reflect possible increased exposure or differential accumulation and is consistent with studies observing greater abundance of parabens in urine of other estrogen-mediated breast cancer patients^9,43,49^.

We also correlated relative levels of methyl-4-hydroxybenzoate sulfate with other metabolites detected in the cervicovaginal lavages, including other xenobiotics. This analysis was performed because parabens may be present as excipients in certain medications, and medication use patterns may differ between healthy individuals and cancer patients. In the cervical cancer cohort, methyl-4-hydroxybenzoate sulfate strongly correlated with the parent molecule 4-hydroxybenzoate and metabolite phenol sulfate (Spearman’s ρ = 0.88, and 0.81, respectively). It also correlated with several carnitine metabolites (i.e., myristoylcarnitine (Spearman’s ρ = 0.86), laurylcarnitine (Spearman’s ρ = 0.83), which were metabolic signatures of cervical cancer^46^, but also with a particular pharmaceutical, naproxen (Spearman’s ρ = 0.73) and its liver metabolite desmethyl-naproxen sulfate (Spearman’s ρ =0.71). Naproxen formulations, particularly oral suspensions, contain methyl-paraben to extend shelf-life. The same analysis on the endometrial cancer study demonstrated a weak correlation between naproxen and methyl-4-hydroxybenzoate (Spearman’s ρ =0.14). The differences observed between the two cohorts could potentially be due to differences in their respective control groups; the cervical cohort included individuals with no apparent health conditions, whereas the endometrial cohort controls consisted of individuals with benign gynecological conditions. Overall, our data demonstrated the presence of parabens (and/or their liver-transformed metabolites) in the local cervicovaginal microenvironment of patients recently diagnosed with cervical or endometrial cancer, expanding previous findings that were largely limited to measurements in systemic matrices such as blood, urine, and other peripheral biospecimens. As demonstrated by our analysis, examining the correlation between drug use and hepatic metabolism of parabens highlights the importance of considering pharmaceutical-associated exposure when evaluating potential biomarkers.

### Moving from co-occurrence to causation: potential mechanisms between paraben metabolites and hallmarks of estrogen-mediated cancers

To investigate the causative role of paraben exposure on the etiology of estrogen-mediated cancers, several cell culture models have been repurposed or developed. These experimental models provide different levels of biological and translational insights. Immortalized cell lines are useful for investigating molecular pathways under controlled conditions; primary human cells may better reflect human tissue physiology; and animal models provide organism-level evidence but do not fully recapitulate human exposure patterns, hormonal environments, or gynecologic cancer biology. Similar to the findings from clinical or observational studies, the majority of these models have focused on breast cancer, predominantly using human epithelial mammary gland cell lines. These cell lines were estrogen-receptor (ER) positive or negative and mainly derived from tissues collected from White women, thus not representing ethnic and/or genetic diversity among women. While the majority of the cell lines were immortalized, several studies also included primary cells from women undergoing breast surgery, some derived from Black women^11,50^.

**Fig. 3:**
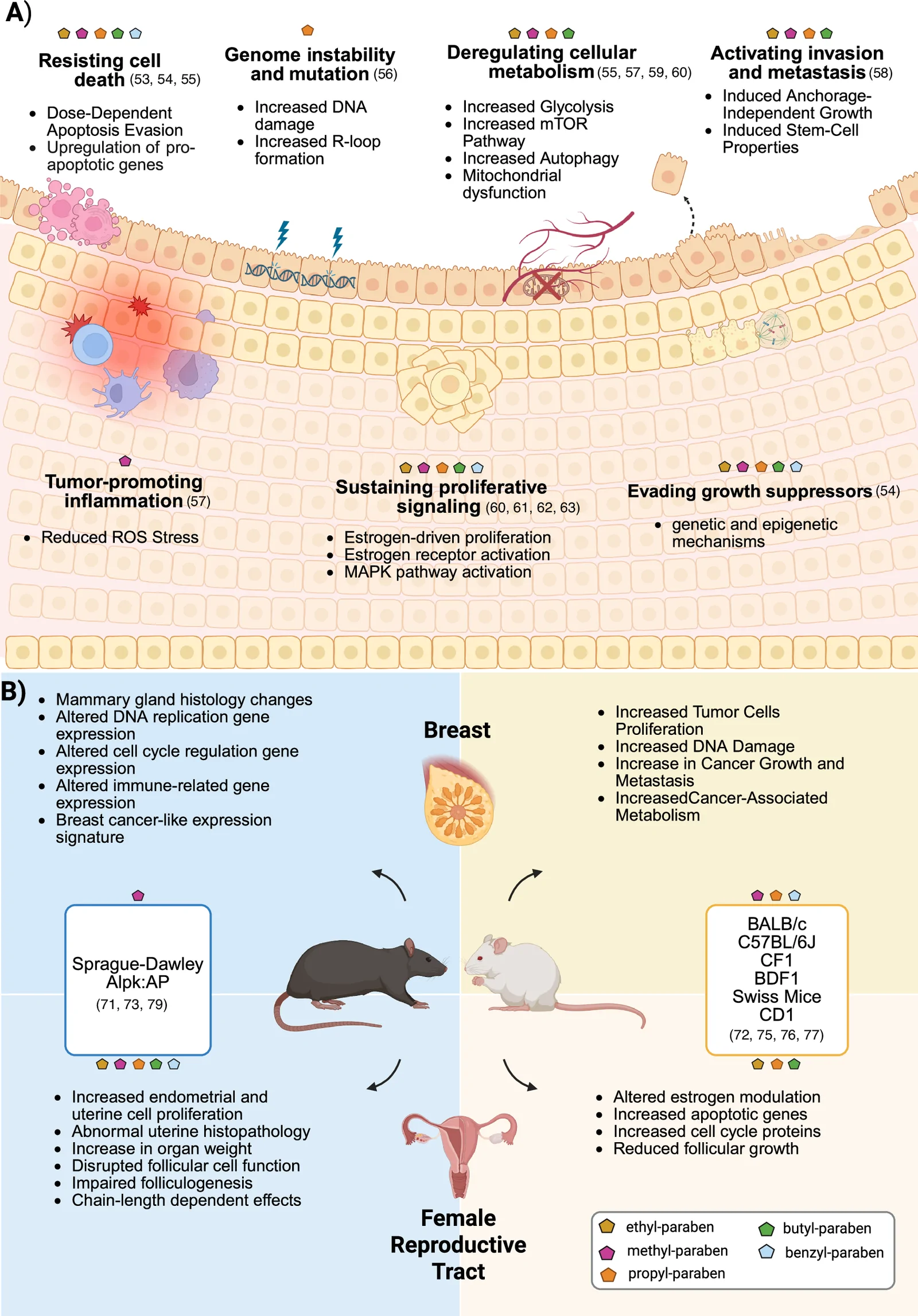
Parabens and hallmarks of cancer in estrogen-mediated cancers. In-vitro (**A**) and in-vivo (**B**) models highlight various mechanisms involved in carcinogenesis of estrogen-mediated cancers in the context of paraben exposures and the outcomes of those on breast and reproductive organs. *Created in BioRender. Herbst-Kralovetz, M. (2025)* https://BioRender.com/rp91efg.

We reviewed several hallmarks of cancer described by Hanahan and Weinberg^51^ including, sustaining proliferative signaling, deregulating cellular metabolism, resisting cell death, activating invasion and metastasis, evading growth suppressors, tumor proliferating inflammation, and genome instability and mutation, to better understand the mechanisms by which parabens could be influencing carcinogenesis (Fig. 3A). These mechanisms can be impacted through estrogen receptor signaling^52^ or via independent mechanisms. Among the hallmarks of cancer, paraben esters induced apoptosis evasion^53^ in a dose-dependent manner^54^; however, transcriptomic profiling also indicated that parabens upregulated pro-apoptotic genes^54,55^ in the immortalized MCF-10A cell line. There was also evidence of genotoxicity; for example, propyl-paraben was shown to induce DNA damage in both immortalized MCF7 and T47D breast tumor epithelial cell lines^56^. Interestingly, parabens did not seem to induce cytokine production or other inflammatory markers in the tested epithelial cell lines (both primary and immortalized), although these in vitro studies did not test cytokine production by immune cells following exposure to parabens. However, reduced ROS stress with methyl-paraben^57^ linked to tumor-promoting inflammation in primary breast cancer cells has been observed. Long-term exposure (=17 days) to parabens contributed to migration and anchorage-independent growth in immortalized MCF-10A mammary gland tumor cell lines^58^, representing activation of invasion and metastasis. Parabens impacted several metabolic pathways associated with deregulating cellular metabolism, such as increased glycolysis, mTOR pathway, autophagy, and mitochondrial dysfunction in either primary or immortalized cell lines^55,57,59,60^. Estrogen-driven proliferation and MAPK pathway activation contributing to sustaining the proliferative signaling hallmark were also reported to be activated by various paraben esters^60–63^. Similar to epidemiological studies, the data on parabens and cellular models of gynecological cancers were severely limited. However, studies on primary human granulosa cell models demonstrated that parabens impaired cell growth and mitochondrial function^64^, indicating that paraben exposure may contribute to hallmarks of ovarian cancer, and these experimental findings support mechanistic plausibility.

Besides the reproductive and breast cell models, interactions between parabens and immune cells have been investigated under in vitro conditions, given that immune dysregulation and chronic inflammation are well-established hallmarks and enabling characteristics of cancer. For example, at physiologically relevant concentrations, five common paraben esters exhibited immunotoxic effects on macrophages by increasing phagocytosis while dampening chemotaxis and secretion of major pro-inflammatory cytokines (TNF-α and IL-6) as well as anti-inflammatory IL-10^65^. Similarly, neutrophils isolated from human donors that were exposed to methyl-paraben demonstrated reduced phagocytic capacity, chemotaxis, and superoxide amine production^66^, suggesting altered innate immune responses and impaired immune surveillance. In parallel, parabens enhanced retinoic acid-related orphan receptor γt-dependent transcription of pro-inflammatory IL-17A and IL-22 genes in a murine Th17 cell model^67^, indicating potential modulation of inflammatory T-cell pathways. These interactions suggest that parabens may influence immune pathways relevant to tumor-promoting inflammation and immune regulation; however, the implications for carcinogenesis remain unclear, as activation or suppression of different immune cell populations may exert both tumor-promoting and tumor-suppressive effects depending on the biological context and stage of the disease.

Parabens generally exhibit weaker estrogenic activity and receptor affinity compared to endogenous estradiol^68^, suggesting that their biological effects may be more subtle and context-dependent, particularly in single-exposure studies. However, experimental studies investigating the mechanistic and endocrine-disrupting effects of parabens often employ exposure conditions designed to evaluate molecular pathways and biological responses under controlled settings rather than directly replicating typical human exposure scenarios. At the same time, several studies have examined either long-term exposures (>20 weeks) or a broad concentration range (0.2 nM–10 μM), including physiologically relevant doses (0.2 nM) in breast cancer cell lines^54,69,70^. While these in vitro experimental models provide valuable mechanistic insights into the biological activity of parabens, their translational relevance to chronic human exPposure and cancer risk remains to be fully established.

Animal models (mouse and rat) provided mechanistic evidence between paraben exposure and ovarian follicle or mammary gland health (Fig. 3B). For example, various paraben esters led to increased growth of mammary gland cancer and disruption of ovarian basal follicular cell function or folliculogenesis^71,72^. These findings were consistent across all animal models (C57BL/6J, BDF1 mice or Sprague-Dawley rats)^56,68,73,74^, supporting the outcomes of mechanisms observed from in-vitro cell culture models. Additionally, propyl-paraben disrupted the estrous cycle and reduced oocyte number in BALB/c mice^75^. Whereas prenatal exposure to propyl-paraben induced similar effects on the offspring by disrupting steroidogenesis and accelerated ovarian aging in C57BL/6J mice^76^. Similarly, prepubertal exposure to butyl-paraben led to oxidative stress, disruption of steroidogenesis, and an increase in gonadal weight in Swiss Webster albino mice^77^. These findings highlight that the biological effects of parabens depend on dose, duration, and ester-type. These effects may vary substantially depending on exposure conditions and experimental context. In parallel, findings from developmental and reproductive toxicity studies remain heterogeneous across experimental systems and exposure conditions. While several studies reported endocrine, reproductive, or mammary gland alterations following paraben exposure, other standardized toxicological and multigenerational studies reported limited or no significant adverse reproductive or developmental outcomes^78,79^. Overall, in-vitro and animal models have provided preliminary evidence that paraben exposure can alter cellular function, contribute to hallmarks of cancer and deteriorate organ health. However, findings from experimental models should be interpreted cautiously and may not fully recapitulate human disease biology and exposure patterns.

## Discussion

Emerging evidence suggests a possible association between increased paraben exposure and the occurrence of several estrogen-mediated cancers. However, large gaps in the data hinder definitive conclusions. It remains unclear whether high levels of parabens observed in cancer patients are coincidental—reflecting greater exposure to various endocrine disruptors as exposures occur as cocktails rather than individual compounds—or if they indicate a causal relationship driven by the estrogenic activity of parabens. We highlight several steps that could be taken to move from correlation to causation (Box 1).

Finally, although there are country/region-specific regulations on certain paraben molecules, their widespread production and use remain a global concern. Considering these molecules accumulate in water and soil, meaning their impact on human health extends beyond direct exposure to indirect exposure, is concerning. Strengthening and clarifying the association between paraben exposure and estrogen-mediated cancers will reinforce the need for stricter regulations to limit their inclusion in products as preservatives and encourage the search for safe alternatives. Eventually, it will help consumers make more informed choices, such as opting for drugs, cosmetics, and food items that are paraben-free. Deepening our understanding of how parabens and other endocrine disruptors influence gynecologic and estrogen-mediated cancers is a critical step towards advancing women’s health on a global scale.

Box 1 Actions that can help transition from observed associations between parabens and cancer risk to establishing causal relationships

- Screening larger and more diverse populations, conducting longitudinal studies and establishing biobanks of samples from different biological samples or organs. Longitudinal studies will enable multiple time-point sampling, helping to identify the optimal timing for diagnosis.
- Tracking exposure sources, patterns and frequency of potential environmental exposures in populations prior to cancer diagnosis and throughout treatment.
- Expanding untargeted metabolomic libraries to include a broader range of environmental contaminants, and developing standardized protocols for analyzing various biological specimens.
- Developing personalized or organ-specific models that represent different life stages (for example, prenatal, reproductive age, peri-menopausal or post-menopausal) to relate clinical observations and elucidate underlying mechanisms in humans.
- Investigating estrogen-related mechanisms including parabens’ interactions with estrogen receptors, pathways as well as independent mechanisms in the development and progression of tumorigenesis, and bioaccumulation mechanisms in the cancer tissues.
- Incorporating exposure pathways (for example, geography or lifestyle choices), timing and dosing response schemes, including acute versus chronic exposures.

## Methods

### Search strategy and study selection

PubMed, SCOPUS, Web of Science, and Embase databases were searched. Additionally, Google Scholar was utilized. Our search terms included combinations of “paraben”, “benzoate”, “endocrine-disrupting compounds”, “estrogen-mediated cancer”, “metabolomics”, “breast cancer”, “endometrial cancer”, “ovarian cancer” and “cervical cancer”. Searches were performed between January 2025 and July 2025.

Only articles published in English were considered in this review. Both targeted and untargeted studies were included. Eligible studies comprised observational studies in humans, mechanistic studies using cell culture models (originated from primary or immortalized breast, ovarian, endometrial and cervical cell lines), and experimental studies in animal models. Studies unrelated to cancer, endocrine disruption-related disorders, or paraben-associated mechanisms were excluded unless discussed for contextual relevance. For clinical and epidemiological studies, we extracted information on study design, population characteristics, sample size, biological matrix analyzed, paraben type measured, and analytical method, and key outcomes. For cell culture models or experimental studies, we recorded paraben ester type, model system, study duration and endpoints assessed. Studies were selected based on relevance to the scope of the review and their contribution to understanding exposure, co-occurrence with disease, mechanisms, and evidence approaching causality aspects.

### Metabolome analysis of cervical and endometrial cancer cohorts

Global metabolome data from previously published metabolomic studies on cervical (10 cancer patients and 68 controls including 29 without and 39 with dysplasia)^46^ and endometrial (66 cancer patients and 126 controls including 108 without and 18 with hyperplasia)^47^ cancers were used for an exploratory secondary analysis focused on paraben metabolites. Briefly, in these studies, cervicovaginal lavages (CVL) samples were collected from women who were newly diagnosed with cervical or endometrial cancer and respective control groups, including benign conditions, pre-cancerous lesions, and women with no dysplasia/hyperplasia. Samples were collected from participants who were newly diagnosed with gynecologic cancer prior to any treatment. Untargeted metabolomics was previously performed using ultra-high-performance liquid chromatography-tandem mass spectrometry (UHPLC-MS/MS) as described in detail in the original studies. Metabolite annotations were based on comparison to Metabolon’s authenticated reference library of purified standards, with parameters including retention index, mass-to-charge ratio, and MS/MS spectral matching.

Paraben metabolites were selected a priori as a chemically defined class; relative abundances of 12 benzoate metabolites in the cervical cancer dataset and 20 metabolites in the endometrial cancer dataset were evaluated. Statistical comparisons of metabolite levels across clinical groups were performed using ANOVA, followed by Tukey’s post-hoc test for pairwise comparisons, and significance thresholds were set at *p* < 0.05. Given the small number of pre-specified comparisons and the hypothesis-driven nature of this secondary analysis, *p* values are reported without correction for multiple comparisons. To investigate potential links between paraben metabolites and other detected metabolites, including xenobiotics and pharmaceutical compounds, Spearman’s correlation analysis was conducted. Medication use was inferred from the detected pharmaceuticals in the CVL samples. All analysis were performed using the GraphPad Prism 10 software.

## Supplementary information

Supplementary_Table_1.

## Data Availability

Full and raw data of paraben metabolites in cervicovaginal lavages of women with endometrial and cervical cancer can be found in Supplementary Table S1.
