# Supplementary material for: Parabens and estrogen-mediated cancers: unraveling coincidence, co-occurrence, and causality
Source: NPJ Womens Health. 2026 Aug 28;4(1):31. doi: 10.1038/s44294-026-00162-7 (PMC13637036; doi:10.1038/s44294-026-00162-7)
Supplement: Supplementary file 1 [file 44294_2026_162_MOESM1_ESM.pdf]

Supplementary Table 1. Batch normalized, scaled, and imputed relative intensity values of methyl-4-hydroxybenzoate and benzoate measured in cervicovaginal lavages. Each biochemical was rescaled to set the median equal to 1. Then, missing values were imputed with the minimum.

Methyl-4-hydroxybenzoate sulfate:Endometrial cancer cohort

Benign	Hyperplasia	Endometrial Cancer (EC)
0,145924	0,145924	1,893262
0,493256	0,145924	0,145924
0,145924	0,145924	0,145924
0,145924	0,533405	0,369896
0,145924	1,00966	0,145924
1,563191	0,145924	0,987113
0,145924	1,210644	0,183999
0,145924	2,820123	1,613569
0,145924	0,757015	0,16487
0,145924	0,145924	0,177986
0,145924	2,074389	1,903657
0,154609	2,198848	1,606845
0,145924	0,247749	11,50762
4,96671	1,793925	0,295246
0,173081	0,145924	1,037547
0,145924	0,145924	0,145924
0,145924	0,145924	4,443787
0,145924	0,145924	0,520652
0,145924		0,145924
0,145924		2,97557
0,145924		0,475701
0,145924		0,238887
0,145924		0,331693
0,225572		1,060196
0,962335		2,345561
0,145924		1,711091
0,145924		0,145924
0,145924		0,270286
0,145924		0,145924
0,99034		0,346424
0,145924		2,617098
0,170012		0,693662
2,303996		0,145924
1,891221		0,145924
8,735679		2,761319

Methyl-4-hydroxybenzoate sulfate: Cervical cancer Cohort

Control	Dysplasia	Cervical cancer (ICC)
1,5101	0,2604	0,2604
0,2604	0,2604	0,2902
0,2604	1,4344	10,7252
0,2604	0,544	0,2604
0,2604	0,2604	15,2023
0,2604	0,2604	0,2604
0,2604	0,2604	34,5074
0,2604	0,2604	0,2604
0,3008	0,2604	1,5379
0,2604	0,2604	2,0847
0,2604	0,2604	
0,2604	0,2604	
0,2604	0,2604	
0,2604	0,2604	
0,2604	0,2604	
1	0,4049	
0,2604	0,2604	
0,2604	0,2604	
0,2604	0,2604	
0,2604	0,2604	
1,9862	0,2604	
0,2604	0,2604	
0,2604	0,2604	
2,4818	0,2604	
0,4275	0,2604	
0,2604	0,2604	
0,2604	0,8841	
0,2604	0,2604	
0,5355	0,2604	
	0,2604	
	0,2604	
	0,2604	
	1,4769	
	0,2604	
	0,2851	

Benzoate: Endometrial cancer cohort

Benign	Hyperplasia	Endometrial Cancer (EC)
1,62208	0,09341	1,100988
0,09341	0,09341	0,602172
1,90381	0,152434	2,632739
0,74587	0,588429	2,884386
0,80714	1,005019	0,988476
2,01594	0,742077	1,33533
0,09341	0,589282	0,334485
0,95946	0,63114	0,09341
1,20075	0,69201	1,294402
0,13302	0,589068	2,084846
0,09341	0,744743	0,631732
0,09341	0,334413	3,182016
2,5484	0,520522	1,498497
3,79886	0,926346	0,589316
0,5457	1,942279	3,628005
0,09341	0,241291	2,58534
0,09341	1,937797	3,474551
0,98092	0,09341	1,310475
1,48502		2,446453
0,09341		0,09341
0,26411		0,388661
0,09341		0,09341
0,49802		1,10048
0,46179		1,426318
1		0,385146
0,09341		2,620506
1,94269		2,574005
0,09341		0,342831
0,09341		0,892351
0,32438		1,472984
0,412		3,097956
0,28877		2,50708
0,09341		0,09341
0,5925		5,016491
2,94907		2,955409

0,145924		0,145924
0,145924		0,145924
0,145924		2,130509
0,145924		6,420333
0,627778		0,651099
0,145924		0,145924
0,173548		0,145924
0,145924		0,145924
1,545644		0,655604
0,145924		0,145924
0,145924		0,145924
0,145924		3,17575
0,744057		0,410323
0,145924		0,650445
1,517163		0,145924
0,145924		4,085076
0,145924		0,145924
0,145924		1,091707
0,145924		2,727704
1,462995		0,242882
0,145924		0,20384
0,145924		0,145924
0,145924		5,292596
0,145924		0,145924
0,145924		2,44192
0,145924		0,145924
0,351969		0,145924
0,145924		5,064372
0,145924		8,230575
0,145924		5,920663
0,145924		0,388741
0,145924		
0,172456		
19,40457		
0,736691		
0,145924		
0,145924		
1,989095		
0,25068		
0,413106		
0,145924		
0,145924		
0,145924		
0,145924		
0,145924		
0,145924		

	0,6572	
	0,2604	
	0,2604	
	0,2604	

0,44955		1,730106
0,09341		5,079984
0,09341		2,077321
0,09341		2,393623
0,48938		1,641675
0,81389		1,947581
1,17325		1,638172
0,19109		0,645617
0,50209		0,915851
0,09341		0,435988
0,09341		0,841467
0,09341		1,669949
1,66945		0,172136
0,57135		1,516044
1,0982		1,676862
0,09341		1,074965
0,71581		0,409304
1,87153		1,875458
0,09341		2,851249
0,44315		1,174355
0,09341		2,311455
1,5502		0,467723
0,09341		5,169778
0,2721		0,302417
0,09341		2,123693
1,0334		1,569765
1,35939		0,862506
2,91669		1,335839
0,09341		1,197305
0,09341		2,085661
0,86117		0,766094
0,09341		
0,28412		
3,06315		
0,40908		
0,09341		
0,60877		
0,48969		
0,09341		
0,51651		
1,29391		
0,09341		
0,10289		
0,76353		
0,09341		
0,09341		

1		
0,145924		
0,145924		
0,145924		
0,243565		
0,72274		
0,145924		
0,145924		
0,145924		
0,145924		
0,145924		
0,145924		
0,145924		
0,595644		
0,145924		
2,091383		
0,145924		
0,145924		
0,145924		
0,145924		
1,177527		
2,113791		
0,243837		
0,145924		
0,145924		
0,145924		
0,145924		

0,09341		
0,09341		
0,09341		
0,09341		
0,16821		
0,96768		
0,09341		
0,12242		
0,09341		
0,09341		
0,35266		
0,09341		
0,23346		
0,09341		
0,09341		
1,13537		
0,09341		
0,09341		
0,09341		
0,9856		
0,45801		
1,0144		
1,29887		
0,09341		
3,41025		
1,56519		
0,3185		
